# Genomic mosaicism in colorectal cancer and polyposis syndromes: a systematic review and meta-analysis

**DOI:** 10.1007/s00384-024-04776-8

**Published:** 2024-12-15

**Authors:** Francisco Cezar Aquino de Moraes, Nayara Rozalem Moretti, Vitor Kendi Tsuchiya Sano, Cristiane Wen Tsing Ngan, Rommel Mario Rodríguez Burbano

**Affiliations:** 1 Federal University of Pará, Belém, Pará, 66073-005 Brazil; 2 University of Western São Paulo, Presidente Prudente, 19050-920 Brazil; 3 Federal University of Acre, Rio Branco, Acre, 69920-900 Brazil; 4 University Anhembi Morumbi, Piracicaba, São Paulo, 13425-380 Brazil; 5 Ophir Loyola Hospital, Belém, Pará, 66063-240 Brazil

**Keywords:** Colorectal cancer, Mosaicism, Polypoid syndromes, APC mutation, MLH1, MLH1, MSH2

## Abstract

**Background:**

Colorectal cancer (CRC) and polypoid syndromes are significant public health concerns, with somatic mosaicism playing a crucial role in their genetic diversity. This study aimed to investigate the prevalence and impact of somatic mosaicism in these conditions.

**Methods:**

A search was conducted using PubMed, Scopus, and Web of Sciences to identify studies evaluating mosaicism in patients with CRC or polyposis syndromes. Odds ratios (ORs) with 95% confidence intervals (CIs) were calculated to determine prevalence rates. Statistical analyses were performed using R software 4.3.

**Results:**

A total of 27 studies, encompassing 2272 patients, were included in the analysis. Of these, 108 patients exhibited somatic mosaicism, resulting in an overall prevalence of 8.79% (95% CI 5.1 to 14.70%, *I*^2^ = 85; *p* < 0.01). Subgroup analyses revealed a significantly higher prevalence of mosaicism in patients with APC mutations (OR 13.43%, 95% CI 6.36 to 26.18%, *I*^2^ = 87; *p* < 0.01). Additionally, mosaicism in MLH1 and MSH2 genes was observed at rates of 2.75% (95% CI 1.20 to 6.18%) and 9.69% (95% CI 2.98 to 27.24%), respectively.

**Conclusions:**

Our findings support the growing recognition of mosaicism as a critical factor in CRC susceptibility and underscore the importance of incorporating mosaicism screening into routine genetic testing for at-risk patients.

**Supplementary Information:**

The online version contains supplementary material available at 10.1007/s00384-024-04776-8.

## Introduction

Colorectal cancer (CRC) is a serious public health issue, with approximately 1.9 million new cases diagnosed annually [1–5]. Many patients are diagnosed at advanced stages, which limits treatment options and reduces survival rates [6]. Understanding genetic factors, such as somatic mosaicism, that influence the development of CRC is essential for improving early detection and personalizing treatments [7, 8]. Somatic mosaicism, which involves the presence of genetically distinct cells within the same individual, is a significant factor in the genetic diversity of diseases like CRC and polypoid syndromes [9–11]. This mosaicism can arise both early in development and later in life, affecting the severity and progression of the disease in various ways [12, 13].

During embryogenesis, the timing of a mutation determines its distribution and impact on the organism [14–16]. If a mutation occurs shortly after fertilization, during the first cell divisions, it may be incorporated into several germ layers of the embryo, such as the ectoderm, mesoderm, and endoderm [12, 17, 18]. This means that the mutation could be present in various tissues and organs, resulting in a more widespread phenotype, which could lead to the development of polyps in different regions of the colon and affect other organ systems, increasing the complexity and severity of the clinical condition [19, 20]. On the other hand, if the mutation occurs at a later stage of embryogenesis, when the cells are more differentiated, its impact will be more localized [15, 21]. This could result in a more restricted manifestation of the disease, such as the formation of polyps in a single region of the colon [19]. This temporal differentiation is crucial for understanding the variations in the clinical presentation of CRC and polypoid syndromes in patients with somatic mosaicism [10, 22].

Somatic mosaicism, characterized by the presence of genetically distinct cell populations within an individual, is increasingly recognized as a significant contributor to the genetic diversity observed in various diseases, including CRC and polypoid syndromes [9, 23–25]. In colorectal cancer, somatic mosaicism may manifest as early mutations that set the stage for the development of both benign and malignant lesions [26, 27]. For instance, mutations in genes such as APC, which play a critical role in the WNT signaling pathway, are often among the first events in the adenoma-carcinoma sequence [28–30]. When these mutations occur in a mosaic pattern, different regions of the colon may harbor distinct genetic alterations, leading to varying degrees of polyp formation and cancer risk within the same individual [11, 31–33]. Polypoid syndromes, such as familial adenomatous polyposis (FAP) and Lynch syndrome, may also exhibit mosaicism [10, 34]. In FAP, mosaicism in the APC gene can result in a milder phenotype, with fewer polyps and a later onset of cancer compared to individuals with germline mutations [35, 36]. This variation can complicate diagnosis and management, as standard screening protocols may not fully capture the extent of the disease in cases of mosaicism [12, 37].

Understanding the role of somatic mosaicism in CRC and polypoid syndromes is crucial for improving diagnosis and treatment. The presence of mosaicism can complicate diagnosis and require more personalized strategies for clinical management. Therefore, this meta-analysis seeks to clarify the role of somatic mosaicism in CRC and polypoid syndromes, as well as to characterize the frequency of this condition in this patient group.

## Methods

### Protocol and registration

This systematic review and meta-analysis were conducted following Preferred Reporting Items for Systematic Reviews and Meta-Analysis (PRISMA) guidelines and the recommendations from the Cochrane Collaboration, as detailed in Supplementary Material: Tables 1 and 2 [38]. The study was officially registered in the Prospective International Registry of Systematic Reviews (PROSPERO) under the identifier CRD2344534945 and is accessible at https://www.crd.york.ac.uk/ as of 31 July 2024 [39].

### Eligibility criteria

Studies that adhered to the following eligibility criteria were considered: (1) retrospective cohort studies, (2) case–control studies, (3) observational studies, (4) studies that provided data on patients who were officially diagnosed with colorectal cancer or polyposis syndromes, and (5) studies with patients who underwent testing for mosaicism. Articles that did not include confirmed diagnostic data for colorectal cancer or polyposis or lacked mosaicism testing were not considered. Moreover, studies with study designs such as case reports, reviews, opinion pieces, technical reports, guidelines, animal studies, and in vitro experiments were also excluded. Only studies published in English were considered, and the publication date was not restricted.

### Search strategy

A systematic search of published studies was conducted on PubMed, Scopus, and Web of Science in August 2024. The search was further extended to include abstracts, articles, and scientific presentations. For each database, search strategies were meticulously adapted using both Medical Subject Headings (MeSH) and input terms, following the specific syntax rules of each platform. Boolean operators (OR, AND) were employed to effectively combine search terms. To broaden our review’s scope, we also examined the references of the included articles and relevant systematic reviews. Concurrently, we ensured the currency of our research by setting up alerts in each database to notify us of newly published studies relevant to our search criteria. The detailed search strategies are provided in Supplementary Table 3.

The search strategy was executed collaboratively by two authors (N.R.M. and F.C.A.M.). To ensure comprehensive coverage, we assessed the included articles’ references and abstracts and conducted systematic literature reviews. All studies screened through databases and references were imported into the reference management software (Rayyan version 1.1). Duplicates were removed using automatic screening (Zotero® version 6.0.37; Thomson Reuters, Philadelphia, PA, USA) and manual review. The titles and abstracts of the identified articles were independently reviewed by the two authors, who also independently extracted data according to predefined search criteria and quality assessment protocols. In cases of discrepancy between the reviewers, a third reviewer provided the final decision on study inclusion.

### Data extraction and risk of bias assessment

To compile the principal outcomes, two authors (N.R.M. and F.C.A.M.) independently extracted analyzed data from each included article. The variables collected encompassed the primary author and year of publication, the overall patient cohort size, the specific type of patients tested, the number of mosaic patients identified, the type of mosaicism detected, the methodologies employed for detection, and detailed patient phenotypes, including the number of individuals affected by each phenotype.

The Newcastle–Ottawa Scale (NOS) was used to assess the risk of bias and quality of the included studies. The scale evaluates studies across 7 to 8 domains depending on the study type, with each domain rated as “low risk,” “unclear risk,” or “high risk.” Studies were categorized based on total scores, with those scoring ≥ 7 considered high quality and those scoring < 7 regarded as low quality. The NOS allocates points based on key criteria such as the selection of study cohorts, the comparability of groups concerning critical factors, and the assessment of outcomes, including follow-up duration and adequacy [40]. Two reviewers (N.M.R. and F.C.A.M.) conducted evaluations independently, ensuring objectivity and reducing bias. Discrepancies between assessments and any conceptual, methodological, or statistical issues were resolved through consensus discussions involving the research team. Funnel plots were utilized to analyze the symmetry of all outcomes.

### Endpoints and definitions

The primary outcomes of interest in this systematic review and meta-analysis were as follows: (1) determine the prevalence of mosaicism in patients diagnosed with colorectal cancer or polyposis syndromes; (2) identify the different types of mosaicism (APC, MLH1, and MSH2) present associate with the phenotype.

### Statistical analysis

Baseline characteristics of the sample were analyzed to assess their potential impact on the outcomes. Prevalence rates were calculated, providing 95% confidence intervals (CIs) for each outcome. A fixed-effect model was applied for low heterogeneity outcomes (*I*^2^ < 25%) [41]. The DerSimonian and Laird random-effects model was employed for those with significant heterogeneity to account for variability across studies [42]. Heterogeneity and effect sizes were quantified using *I*^2^ and Tau^2^ statistics. All statistical analyses were conducted using R statistical software, version 4.2.3 (R Foundation for Statistical Computing). To ensure the robustness of the findings, sensitivity analyses were performed using leave-one-out and funnel plots.

## Results

### Study selection and baseline characteristics

**Fig. 1:**
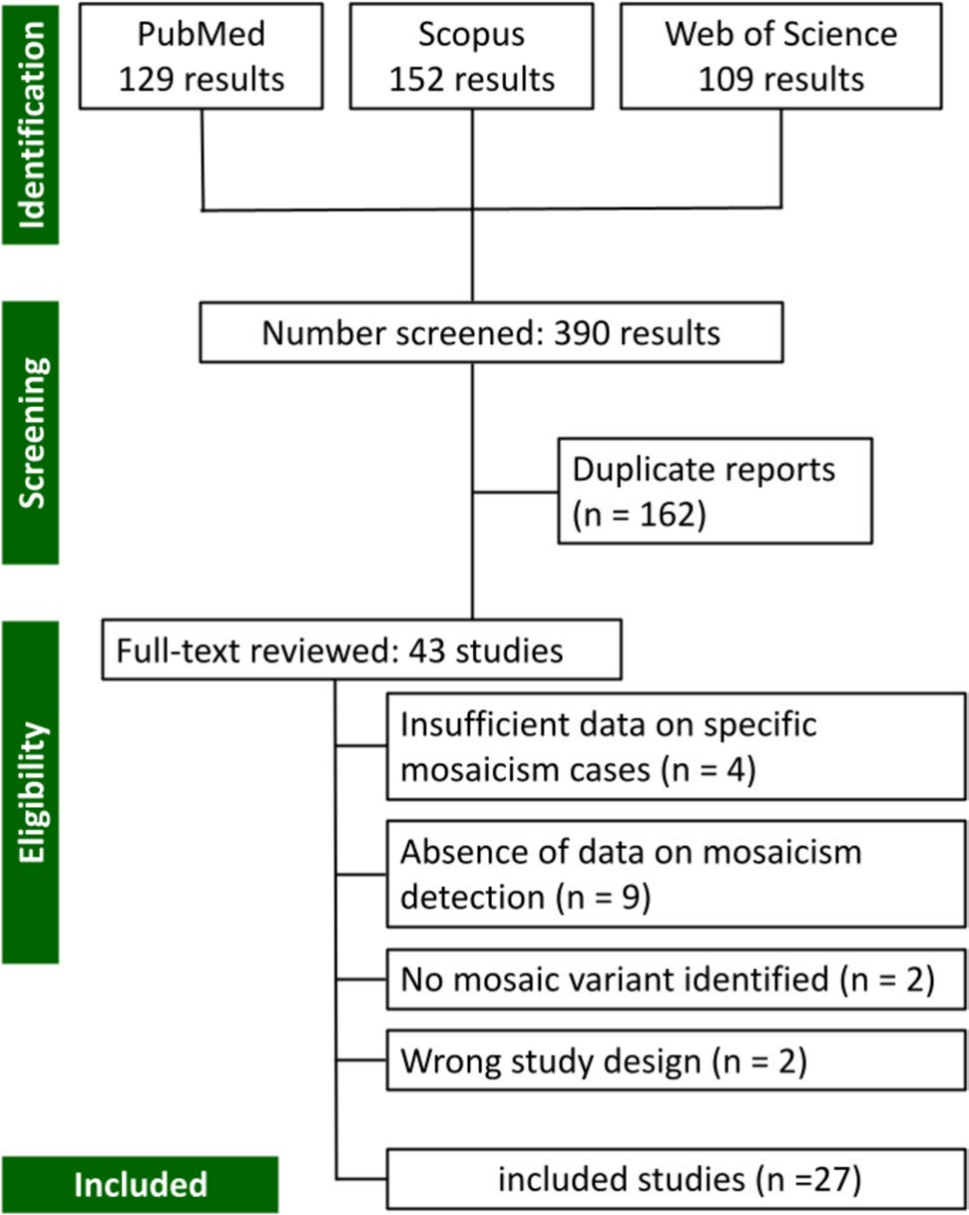
Flowchart of studies included

Figure 1 illustrates the study selection process for the meta-analysis. Initially, 390 results were identified from three databases: PubMed (129 results), Scopus (152 results), and Web of Science (109 results). After the initial screening, 162 duplicate studies were removed, leaving 43 studies for full-text review. Of these, 16 studies were excluded for the following reasons: 4 due to insufficient data on specific mosaicism cases, 9 due to the absence of data on mosaicism detection, 2 because no mosaic variants were identified, and 2 for having the wrong study design. In total, 27 studies were included in the meta-analysis.

### Characteristics of the included studies

A total of 27 studies were included, involving 2272 patients, of which 108 had mosaicism. Fourteen studies found somatic APC, six MLH1, and four MSH2. The genotyping method most used was next-generation sequencing (NGS). These characteristics are detailed in Table 1.

**Table 1:** Characteristics of the included studies

Author, year	Type of patients tested	Mosaic patients	Patients tested	Type of mosaicism	Detection method	Patient phenotypes (no. of patients affected)
Aretz, 2007 [43]	Suspected or confirmed de novo APC mutation	8	75	Somatic APC	PTT, DHPLC, SNaPshot	AFAP (5), FAP 100–200 (3)
Baert-Desurmont, 2018 [44]	Patients with suspected hereditary colorectal cancer (CRC) and identified with class 4 or 5 genetic variants	2	323	Somatic APC	Sanger, MLPA, NGS	Adenomatous polyposis (1); Diffuse form (2)
		3		Gene STK11		Independent Peutz–Jeghers patients (3)
Bossard, 2012 [45]	Patients with metastatic colorectal adenocarcinoma (mCRC)	4	18	KRAS Gene	PCR	CRC (4)
Chan, 2006 [46]	Early-onset or familial MSI CRCs	1	31	Gene MSH2	MSP, Clonal Bisulfite Sequencing, haplotype analysis, pyrosequencing	CRC < 50 (2) EC < 50 (1) Adenomas > 70 (1) No phenotype (6)
Ciavarella, 2018 [47]	Patients with unexplained colorectal adenomatous polyposis	4	8	Somatic APC	Sanger, Dpcr, WES	AFAP (2), FAP 100–200 (2)
Farrington, 1999 [48]	Parents of de novo probands	2	5	Somatic APC	Sanger, PCR cloning, single cell analysis	FAP (2)
Guillerm, 2020 [49]	Patients with Lynch-like syndrome with mismatch repair gene mutations (MMR)	1	15	Gene MSH2	NGS and Sanger	CRC
Hes, 2008 [50]	APC mutation carriers	10	242	Somatic APC	DGGE/PTT	No polyps (1), AFAP(4), FAP (5)
Hitchins, 2011 [51]	Patients with early-onset CRC and a LS-like phenotype with negative mismatch repair gene mutations (MMR)	1	122	Gene MLH1	qMSP, COBRA	CRC < 50
Hitchins, 2023 [52]	Patients with early-onset CRC and a LS-like phenotype with mismatch repair gene mutations (MMR)	4	281	Gene MLH1	Pyrosequencing, PCR and Clonal Bisulfite Sequencing	CRC
Jansen, 2016 [53]	Patients with unexplained adenomatous polyposis or multiple primary colorectal carcinomas, who tested negative for germline APC and MUTYH mutations	9	18	Somatic APC	NGS, Sanger	AFAP (9)
Joo, 2023 [54]	Patients with early-onset CRC and suspected MLH1 epimutation	3	97	Gene MLH1	ddPCR	CRC (2); EOCRC—Early-Onset Colorectal Cancer (1)
Kanter-Smoler, 2008 [55]	APC- and MUTYH-negative patients with de novo mutations	1	3	Somatic APC	SSCP	AFAP
Karstensen, 2024 [56]	Patients with AFAP and negative for known pathogenic variants in common polyposis-associated genes	2	27	Somatic APC	NGS	AFAP (2)
Kim, 2019 [57]	Patients with clinically suspected familial adenomatous polyposis (FAP) who had no detectable pathogenic variants in known colonic polyposis-associated genes	7	28	Somatic APC	NGS and MEMO-PCR	FAP
Mongin, 2012 [58]	De novo FAP patients without family history	1	17	Somatic APC	HRM, NGS, and Sanger	FAP
Morak, 2008 [59]	Patients with suspected HNPCC (hereditary nonpolyposis colorectal cancer) or Lynch syndrome, who had MSI-H tumors and loss of MLH1 protein expression, but tested negative for germline mutations in MMR genes, with 12 of them showing aberrant MLH1 promoter methylation	6	94	Gene MLH1	MSP, MS-MPLA, SNP typing, haplotype analysis	CRC (6)
Mur, 2014 [60]	LS-suspected families	6	22	Gene MSH2	MLPA, MS-MLPA	CRC < 50 (7), CRC52 (1), DC52 (1) Hg.ad28 (1)
Out, 2015 [61]	Unexplained AFAP patients	4	173	Somatic APC	HRM on leukocyte (171) HRM on tumor DNA (2)	AFAP (4)
Pinto, 2018 [62]	Mutation negative polyposis or CRC patients	4	38	Gene MLH1	MS-MLPA, qMSP, ddPCR	CRC < 50 (4)
Rofes, 2021 [63]	Patients with classic familial adenomatous polyposis (FAP) who had no causative germline variants identified in the APC and/or MUTYH genes	7	11	Somatic APC	NGS and Sanger	FAP (7)
Sourrouille, 2013 [64]	Patients with microsatellite instability (MSI) colorectal cancer, suspected of having Lynch Syndrome, who tested negative for germline mutations and promoter methylation in MMR genes, specifically focusing on those with loss of MSH2 protein expression	1	18	Gene MSH2	Sanger, MLPA, HRM	CRC
Spier, 2016 (NGS) [65]	Unexplained Types of mosaicism included variants detected in leukocytes FAP patients > 20 synchronous adenomas > 40 non-synchronous adenomas)	5	20	Somatic APC	NGS on adenomas, Sanger and deep sequencing	AFAP (5)
Spier, 2016 (WES) [66]	Unexplained Types of mosaicism included variants detected in leukocytes FAP patients > 40 non- synchronous adenomas)	2	80	Somatic APC	WES on leukocytes, Sanger and deep sequencing	FAP 100–500 (2)
Suter, 2004 [67]	Mutation negative polyposis or CRC patients	2	44	Gene MLH1	COBRA	CRC < 50 (2)
Takao, 2021 [68]	Patients with unexplained colorectal adenomatous polyposis, negative for known germline mutations in 57 genes, including APC and MUTYH	6	46	Somatic APC	High-coverage NGS	FAP (6)
Ward, 2013 [69]	CRC patiens suspected of having Lynch syndrome or displaying early-onset colorectal cancer, but without identified pathogenic germline mutations in the mismatch repair (MMR) genes,	2	416	Gene MLH1	qMSP, MS-HRM analysis, bisulfite sequencing, ddPCR	CRC (2)

* AFAP* attenuated familial adenomatous polyposis, *COBRA* combined bisulfite restriction analysis, *CRC* colorectal cancer, *ddPCR* droplet digital PCR, *FAP* familial adenomatous polyposis, *HRM* high-resolution melting, *MSP* methylation-specific PCR, *NR* not related, *MS-MLPA* methylation-specific multiplex ligation-dependent amplification MS-MLPA, *MLPA* multiplex ligation-dependent amplification, *NGS* next-generation sequencing technology, *PCR* polymerase chain reaction, qPCR quantitative real-time PCR, *qMSP* quantitative methylation-specific PCR, *MEMO-PCR* mutant enrichment with 3′-modified oligonucleotides PCR, *MS-HRM* methylation-sensitive high-resolution melting, *SSCP* single-strand conformation polymorphism, *WES* whole exome sequencing

## General analysis

### Prevalence of overall mosaicism

**Fig. 2:**
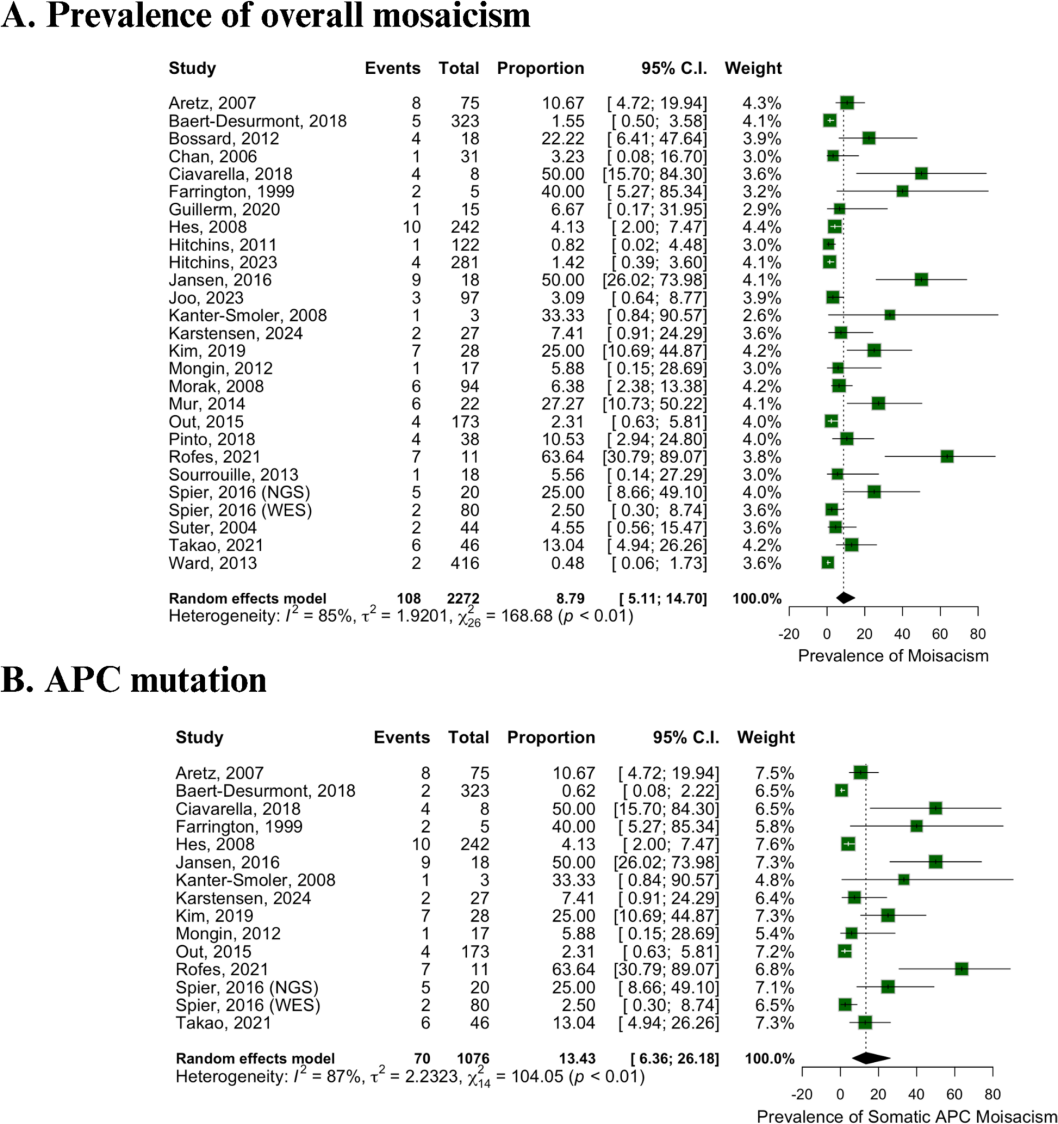
Prevalence. **A** Overall mosaicism. **B** APC mutation

The estimated prevalence of overall mosaicism was determined from 26 studies involving a total of 2,272 patients and 108 events. The prevalence was calculated as an odds ratio (OR) of 8.79% (95% CI 5.1 to 14.70%, Fig. 2A). Significant heterogeneity (*I*^2^ = 85; *p* < 0.01) was observed, which is expected due to the inclusion of observational studies.

### APC mutation

The prevalence of APC mutations in patients with gastric cancer or polyposis syndromes was assessed in 15 studies encompassing 1076 patients. Seventy APC mutations were identified, resulting in an estimated OR of 13.43% (95% CI 6.36 to 26.18%, Fig. 2B). The heterogeneity for APC mutations was also significant (*I*^2^ = 87; *p* < 0.01).

### MLH1 and MSH2 mosaicism

**Fig. 3:**
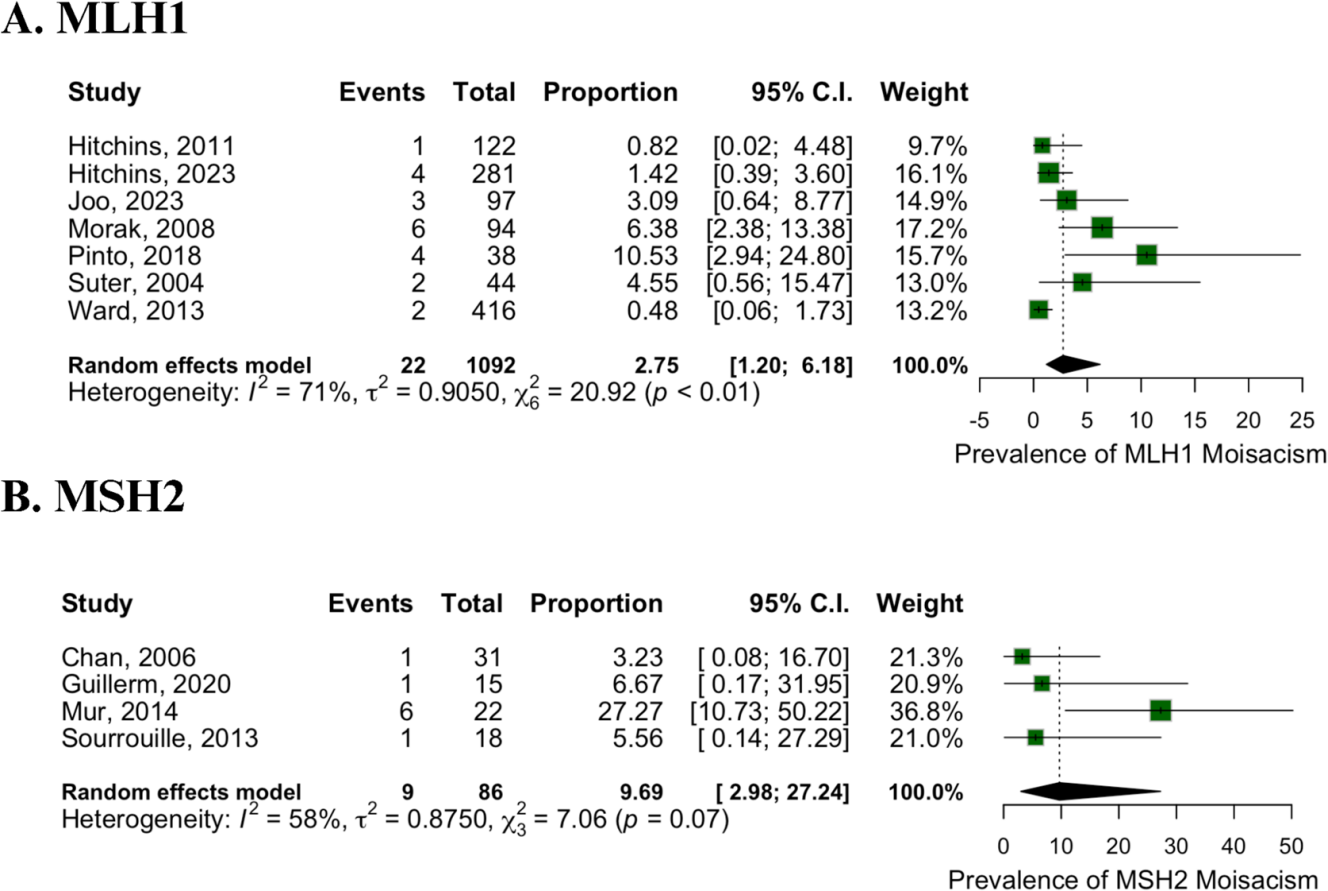
Prevalence. **A** MLH1. **B** MSH2

The prevalence of MLH1 mosaicism was analyzed in 7 studies involving 1092 patients. Twenty-two cases of MLH1 mosaicism were identified, leading to an estimated prevalence of 2.75% (95% CI 1.20 to 6.18%, Fig. 3A). The heterogeneity for MLH1 mosaicism was 71% (*p* < 0.01).

For MSH2 mosaicism, three studies with 86 patients identified 9 cases. The estimated prevalence of MSH2 mosaicism was 9.69% (95% CI 2.98 to 27.24%, Fig. 3B), with a heterogeneity of 58% (*p* < 0.01).

### Sensitivity analysis and quality assessment

**Fig. 4:**
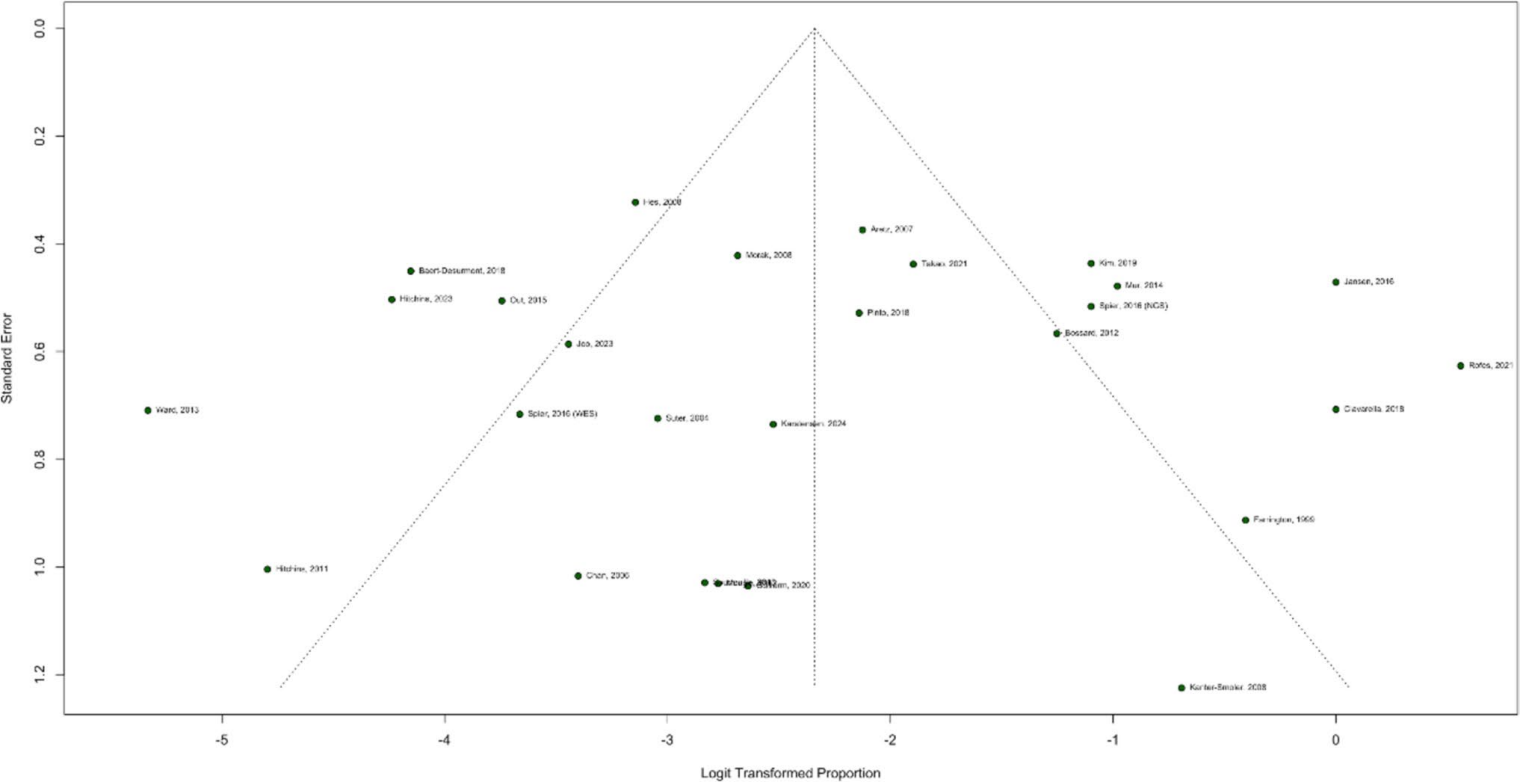
Funnel plot of overall mosaicism

**Fig. 5:**
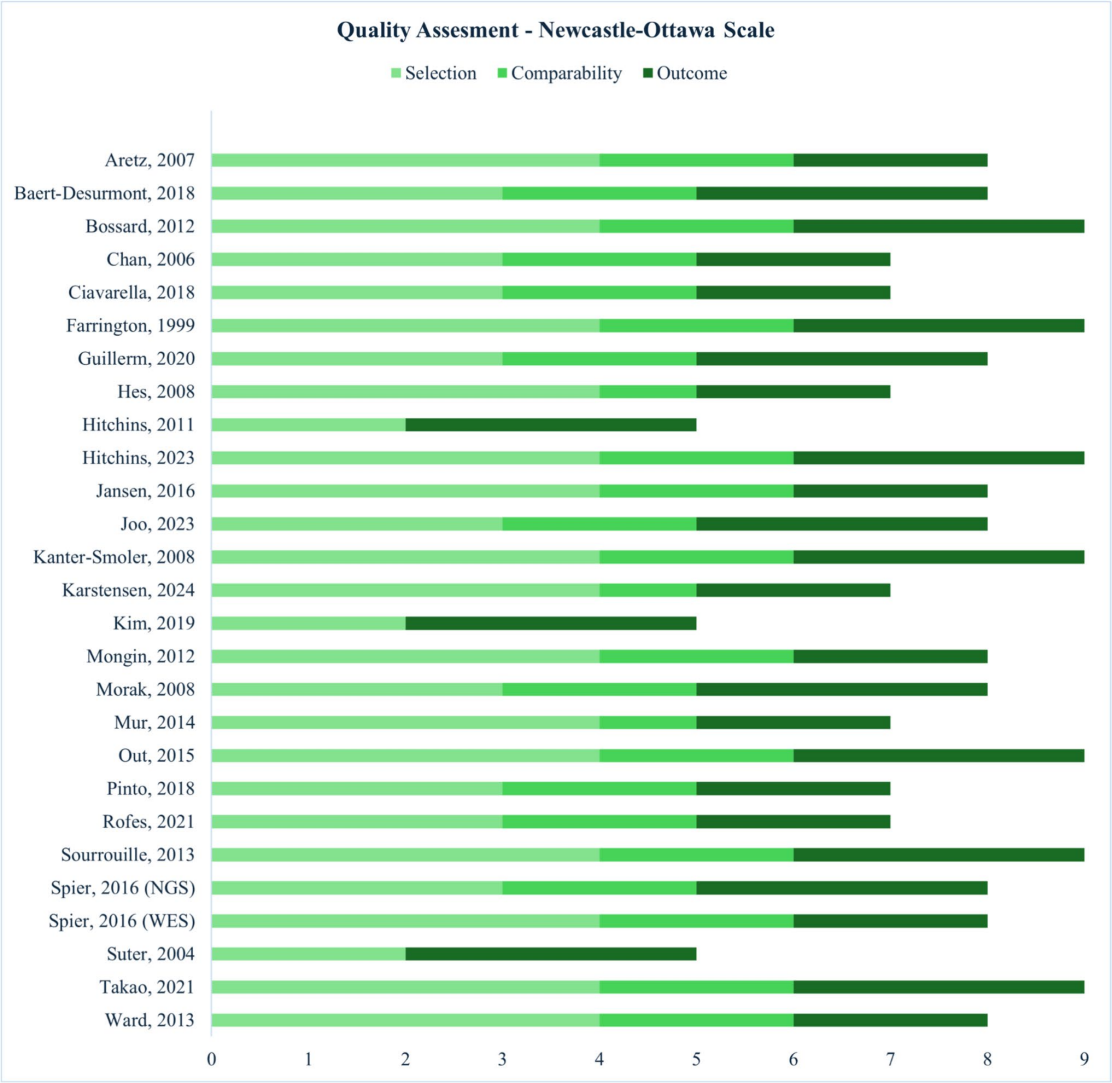
Newcastle–Ottawa scale

A leave-one-out sensitivity analysis was conducted to assess the impact of individual studies on the prevalence estimates of overall mosaicism, APC mutation, MLH1, and MSH2 mosaicism. A significant reduction in heterogeneity (*I*^2^ decreased from 58 to 0%) was observed for the prevalence of MSH2 mosaicism when the Mur 2014 study was excluded. However, omitting individual studies in the other sensitivity analyses did not result in substantial changes to the heterogeneity values. The funnel plot of overall prevalence mosaicism in Fig. 4 exhibited an asymmetrical distribution, suggesting a potential risk of publication bias. This is expected given the nature of the analysis, which involved single-arm observational studies with varying levels of variance. For quality assessment we used the Newcastle–Ottawa Scale for observational studies sixteen studies were considered high quality and eleven studies as low quality (Fig. 5).

## Discussion

This meta-analysis aimed to explore the prevalence and clinical significance of mosaicism in patients with CRC and polyposis syndromes. We identified 27 studies that met our inclusion criteria, with data sourced from major databases, including PubMed, Scopus, and Web of Science. Our systematic review focused on specific genes linked to mosaicism, including APC, MLH1, and MSH2. The findings demonstrate a clear association between mosaicism and these gene mutations, highlighting the importance of mosaicism in CRC pathogenesis and polyposis. The estimated overall prevalence of mosaicism was 8.79%, but this rate varied significantly depending on the gene involved.

Our analysis of APC mutations showed a prevalence of 13.43%, reinforcing the critical role this gene plays in FAP and attenuated familial adenomatous polyposis (AFAP). The high heterogeneity observed in the studies, with an *I*^2^ value of 87%, reflects the complexity of APC mosaicism, which likely arises from its diverse clinical presentations and the range of detection methods used across studies. APC mosaicism is increasingly recognized as a cause of de novo FAP and AFAP, where patients may present with fewer adenomas or a later onset of symptoms [10, 70]. This highlights the need for improved detection techniques to accurately diagnose mosaicism in clinical practice [11, 71, 72].

MLH1 mosaicism, with an estimated prevalence of 2.75%, was less common but still significant. This mutation is associated with Lynch syndrome, a hereditary condition that increases the risk of CRC and other cancers [73–75]. Despite its lower prevalence compared to APC mutations, MLH1 mosaicism plays a crucial role in CRC susceptibility [76, 77]. The studies included in this meta-analysis showed moderate heterogeneity (*I*^2^ = 71%), suggesting variability in the methods used to detect mosaic MLH1 mutations. This highlights the need for more standardized protocols in future research to accurately assess the prevalence and clinical implications of MLH1 mosaicism [78].

The prevalence of MSH2 mosaicism, reported at 9.69%, was based on three studies involving 86 patients. Although the sample size was smaller, the heterogeneity was relatively low (*I*^2^ = 58%), indicating a more consistent detection method across these studies. MSH2 mosaicism, like MLH1, is associated with Lynch syndrome and poses a significant risk for CRC development [73, 79–81]. Given the increasing recognition of mosaic mutations in Lynch syndrome genes, it is essential to incorporate mosaicism screening into the standard genetic testing for patients with suspected Lynch syndrome, particularly when no germline mutations are identified [75, 82].

The overall prevalence of mosaicism in our analysis, 8.79%, may be an underestimate due to the limitations in detection methods, particularly in older studies that relied on less sensitive techniques. Newer methods, such as NGS and droplet digital PCR, have shown greater sensitivity in detecting low-level mosaicism, but their use is not yet widespread in routine diagnostics [83, 84]. This variability in detection technologies likely contributed to the significant heterogeneity observed across studies. Future research should prioritize the development and implementation of more sensitive and standardized methods for detecting mosaicism to provide more accurate prevalence estimates.

One of the major challenges in studying mosaicism is the wide range of clinical presentations, particularly for genes like APC, where mosaic mutations can result in either severe FAP or milder AFAP phenotypes [85]. This clinical variability complicates the diagnosis and management of mosaicism, as traditional genetic screening methods may not capture low-level mosaic mutations present in a small proportion of cells [86, 87]. Our findings emphasize the need for clinicians to consider mosaicism in patients with atypical or milder presentations of polyposis syndromes, even when family history is absent.

The prior study by Jansen and Goel [10], a systematic review on mosaicism in colorectal cancer and polyposis syndromes, identified that genomic mosaicism may play an important role in contributing to the predisposition for colorectal cancer development and in the phenotypic variability observed in hereditary polyposis syndromes. Our analysis expands upon these findings by incorporating additional studies and conducting a meta-analysis, a quantitative assessment of mosaicism frequency and its clinical characteristics within these populations. The statistical analysis in our study not only serves to quantify the frequency of these mutations but is also particularly important for the diagnostic and prognostic implications, which are addressed here for the first time.

The clinical implications of mosaicism extend beyond diagnosis to genetic counseling and patient management. Patients with mosaic variants may have a lower risk of transmitting the mutation to offspring, depending on the timing and extent of the mosaic mutation during embryonic development [11]. However, the potential for germline transmission remains a concern, particularly for early-onset CRC cases [88]. As such, genetic counseling for patients with mosaicism should be approached with caution, and testing of family members may be warranted, especially in cases of APC mosaicism where the risk of transmission is higher.

## Conclusion

In conclusion, this meta-analysis provides important insights into the prevalence and clinical impact of mosaicism in CRC and polyposis syndromes. The significant heterogeneity observed in the studies highlights the need for more standardized detection methods and larger, more comprehensive studies. Despite these limitations, our findings support the growing recognition of mosaicism as a critical factor in CRC susceptibility and underscore the importance of incorporating mosaicism screening into routine genetic testing for at-risk patients. Future research should focus on refining detection techniques and exploring the full clinical spectrum of mosaicism to improve patient care and outcomes.

## Supplementary Information

Below is the link to the electronic supplementary material.

Supplementary file1 (DOCX 1176 KB)

## Data Availability

Availability of data and materials: Data is provided within the manuscript or supplementary information files.
